# The association of high-sensitivity C-reactive protein-triglyceride glucose index with functional outcome in elderly patients with acute ischemic stroke undergoing intravenous thrombolysis

**DOI:** 10.3389/fneur.2026.1913877

**Published:** 2026-09-14

**Authors:** Pengyu Gong, Gang Chen, Shuaiyu Chen, Yaya Fei, Yuye Jiang, Yiyang Zhang, Jiajie Lu, Jianhua Song, Xiaohao Zhang, Ruixiu Zhu, Yuechun Wang

**Affiliations:** 1 Department of Neurology, Affiliated Hospital of Nantong University, Medical School of Nantong University, Nantong, Jiangsu, China; 2 Department of Neurology, Haimen Hospital Affiliated to Nantong University, Nantong, Jiangsu, China; 3 Department of Neurology, Nanjing First Hospital, Nanjing Medical University, Nanjing, Jiangsu, China; 4 Department of Neurology, Nantong Third People's Hospital, Affiliated Nantong Hospital 3 of Nantong University, Nantong, Jiangsu, China; 5 Department of Geriatrics, Nantong Third People's Hospital, Affiliated Nantong Hospital 3 of Nantong University, Nantong, Jiangsu, China

**Keywords:** acute ischemic stroke, elderly, functional outcome, high-sensitivity C-reactive protein-triglyceride-glucose index, intravenous thrombolysis

## Abstract

**Background and purpose:**

High-sensitivity C-reactive protein-triglyceride-glucose index (CTI) is an innovative biomarker of insulin resistance and inflammation. The objective of this study was to explore the association of high-sensitivity CTI with functional outcome in elderly patients with acute ischemic stroke (AIS) undergoing intravenous thrombolysis (IVT).

**Methods:**

Elderly AIS patients treated with IVT were enrolled in three centers. Unfavorable functional outcome was defined as Modified Rankin Scale score ranging from 3–6. Logistic regression models were used to calculate the odds ratio (OR) and 95% confidence interval (95% CI) for the association between high-sensitivity CTI and 3-month functional outcome. Restricted cubic splines (RCS) were performed to explore the shape of this association. Receiver operating characteristic curve was applied to evaluate the discriminatory capacity of high-sensitivity CTI. Furthermore, subgroup and sensitivity analyses were performed to examine the consistency of the observed association.

**Results:**

A total of 708 elderly patients were enrolled, among whom 231 (32.6%) developed unfavorable functional outcome at 3 month follow up. In Model 3, higher continuous high sensitivity CTI levels were independently associated with 3 month unfavorable functional outcome among elderly AIS patients receiving IVT (OR = 1.64; 95% CI, 1.28–2.11). Taking the first quartile of high sensitivity CTI as the reference group, patients in the fourth quartile exhibited the highest risk of unfavorable functional outcome in Model 3 (OR = 2.62, 95% CI, 1.48–4.64). RCS revealed a significant overall association between high sensitivity CTI and unfavorable functional outcome, with no statistically significant nonlinear component (*P* for overall < 0.001; *P* for nonlinearity = 0.743). Subgroup and sensitivity analyses demonstrated consistent directions of associations.

**Conclusion:**

High-sensitivity CTI was independently associated with functional outcome at 3 months in elderly AIS patients treated with IVT.

## Introduction

In China, it is estimated that 14.2% of the population was aged 65 and older in 2021, and this group is anticipated to rise to around 395 million by 2050 (1). Acute ischemic stroke (AIS), which is recognized as a critical health challenge, results in morbidity as well as mortality and imposes a considerable strain on the elderly (2–4). At present, intravenous thrombolysis (IVT) is regarded as an effective treatment for the patients with AIS (5–7). Nevertheless, there still remain numerous elderly AIS patients that may experience unfavorable outcomes even after receiving the treatment of IVT (8, 9). Therefore, it is vital to investigate the biomarkers associated with the clinical outcomes in the elderly patients with AIS undergoing IVT.

Insulin resistance (IR), which contributes to the formation of thrombosis and the progression of atherosclerosis, may play an important role in the ischemic stroke (10, 11). Triglyceride-glucose index (TyG) is reported to be an innovative indicator of IR (12–14). There were several studies that suggested the relationship between TyG and stroke (15–18). What is more, inflammation can serve as a key factor in the pathogenesis of stroke (19–21). Hence, there is a gradual increase in the clinical study focusing on high-sensitivity C-reactive protein-triglyceride glucose index (CTI), which combine TyG, a biomarker of IR, and C-reactive protein (CRP), a biomarker of inflammation (22–24). The results of the study conducted by Hu and his colleagues showed a notable positive linear relationship between the levels of CTI and the occurrence of stroke (24).

In this research, we intended to explore the correlation between the levels of high-sensitivity CTI and functional outcome in the elderly AIS patients treated with IVT.

## Materials and methods

### Study design and participants

Consecutive elderly patients with AIS who received IVT within 4.5 h of symptom onset were prospectively enrolled for data collection and retrospectively analyzed from three participating centers: Nanjing First Hospital, Nantong Third People’s Hospital, and the Affiliated Hospital of Nantong University. All patients were treated in dedicated stroke units. Eligible participants were included in the final analysis according to the following criteria.

Inclusion criteria: 1. Admission within 4.5 h after onset; 2. Treated with IVT; 3. 65 years or older.

Exclusion criteria: 1. Severe inflammatory diseases or infectious diseases within 2 weeks preceding stroke onset; 2. Incomplete data of high-sensitivity CRP or fasting triglyceride or fasting blood glucose; 3. loss to follow-up over the following 3 months after admission.

Informed consent was obtained from participants or their legal representatives. This study was approved by the ethics committees of all participating hospitals.

### Data acquisition

On the day of admission, all the participants underwent standard assessments of demographic characteristics (age and gender), vascular risk factors (history of hypertension, history of diabetes mellitus, history of atrial fibrillation, history of coronary artery disease, current smoking and current drinking alcohol), medication use (antiplatelet and anticoagulation), clinical assessment (stroke severity, onset to treatment time [OTT], proximal arterial occlusion [PAO] and stroke subtype). Computed tomography, magnetic resonance, electrocardiogram, echocardiography, carotid ultrasonography, and transcranial Doppler were performed for assessing stroke subtype and PAO. Stroke severity was assessed using National Institutes of Health Stroke Scale (NIHSS) score. Stroke subtype was classified according to Trial of Org 10,172 in Acute Stroke Treatment (TOAST) criteria (25).

### Measurement of high-sensitivity CTI

The high-sensitivity CTI index was calculated using the following formula: high-sensitivity CTI = 0.412 × Ln (high-sensitivity CRP [mg/L]) + TyG (22, 26–28). The TyG was calculated as Ln (triglyceride [mg/dL] × fasting blood glucose [mg/dL] / 2) (15–18). All samples were obtained within 24 h after stroke onset.

### Definition of outcome

Modified Rankin Scale (mRS), ranging from 0 to 6, is a standard scale to assess the independent status, where higher scores denote poorer independence. To assess functional outcomes in the stroke patients, mRS is widely utilized. At the 3-month follow-up, standardized assessments were performed by certified neurologists via either structured telephone interviews or in-person clinic visits. The primary study outcome was unfavorable functional outcome, which was defined as mRS score ranging from 3–6 (29–31). Meanwhile, favorable functional outcome was defined as mRS score ranging from 0–2 (32–34). What is more, for the purpose of sensitivity analyses, non-excellent functional outcome was termed as mRS 2–6, and excellent functional outcome was termed as mRS 0–1 (35, 36).

### Statistical analysis

Statistical analyses were performed using R version 4.5.0 software.^1^ All participants were categorized into four groups according to the quartiles of high-sensitivity CTI calculated from the present study sample, with cut-off values of 8.743, 9.238, and 9.880 corresponding to Q1-Q4, respectively. Categorical variables were expressed as *n* (%). Group differences across high-sensitivity CTI quartiles (Q1-Q4) were assessed using ANOVA for normally distributed continuous variables, Kruskal-Wallis tests for non-normally distributed continuous variables, and χ^2^ tests or Fisher’s exact tests for categorical variables, as appropriate. We used violin plots to show the distribution of high-sensitivity CTI between the favorable outcome group and unfavorable outcome group. Ordinal logistic regression was used to assess the trend in mRS distribution across CTI quartiles. CTI quartiles were coded as an ordinal numeric variable (1–4) to test the linear trend across quartiles. The proportional-odds assumption was confirmed by the Brant-test for this trend-testing model.

Logistic regression models were performed to estimate associations between the levels of high-sensitivity CTI and unfavorable functional outcome in elderly AIS patients undergoing IVT. Model 1 was the crude model. Model 2 was adjusted for age, gender, current smoking and current alcohol drinking. Model 3 was adjusted for age, gender, current smoking, current alcohol drinking, history of hypertension, history of diabetes mellitus, history of atrial fibrillation, history of coronary artery disease, antiplatelet agents, anticoagulants, NIHSS group, OTT, PAO and stroke subtype.

We further evaluated the trends and strength of the association between the levels of high-sensitivity CTI and unfavorable functional outcome in elderly AIS patients undergoing IVT using restricted cubic splines (RCS) with 3 knots (at the 10th, 50th, and 90th percentiles), adjusted for the covariates in Model 3. Prespecified subgroup analyses were conducted to test the consistency of the association between high-sensitivity CTI and unfavorable functional outcome. Stratified covariates included age, gender, current smoking, current drinking, history of hypertension, history of diabetes mellitus, history of coronary artery disease, and history of atrial fibrillation. The fully-adjusted Model 3 was refitted within each stratum. Between-stratum heterogeneity was evaluated via Z-test of coefficient differences to obtain *p*-values for heterogeneity.

Receiver operating characteristic (ROC) curve analysis was used to test the overall discriminative ability of high-sensitivity CTI for unfavorable functional outcome in elderly AIS patients undergoing IVT. The Youden index was calculated as Sensitivity + Specificity – 1. To test the robustness of our findings, we conducted sensitivity analyses by excluding patients with PAO and assessed the relationships only among patients without PAO. Furthermore, we explored the relationship between the levels of high-sensitivity CTI and non-excellent functional outcome in elderly AIS patients treated with IVT. A two-tailed value of *p* < 0.05 was considered statistically significant.

## Results

### Baseline characteristics

From August 2020 to February 2025, we screened 1,821 AIS patients treated with IVT in this study, initially. A total of 1,113 patients were excluded for the following reasons: 32 patients were excluded for severe inflammatory or infectious diseases; 506 patients were excluded for incomplete data; 278 were excluded for loss to follow-up over the following 3 months after admission. In addition, 297 patients younger than 65 years of age were excluded. There were 708 participants (418 men and 290 women) with a mean age of 75.52 years included in the present study, of whom 231 (32.6%) developed unfavorable functional outcome at 3 months. Table 1 showed the baseline characteristics of the study participants according to the quartiles of high-sensitivity CTI levels. Below are the notable distinctions among these four groups: history of diabetes mellitus (*p* < 0.001), history of hypertension (*p* = 0.037), history of atrial fibrillation (*p* = 0.045), stroke subtype (*p* < 0.001) and functional outcome (*p* < 0.001).

**Table 1:** Characteristics of participants based on the quartile of high-sensitivity CTI.

Characteristics	high-sensitivity CTI				*P*
	Q1 (*n* = 177)	Q2 (*n* = 177)	Q3 (*n* = 176)	Q4 (*n* = 178)	
Age, (%)					0.062
≥75	89 (50.28)	101 (57.06)	89 (50.57)	76 (42.70)	
65–74	88 (49.72)	76 (42.94)	87 (49.43)	102 (57.30)	
Gender, (%)					0.509
Female	72 (40.68)	76 (42.94)	77 (43.75)	65 (36.52)	
Male	105 (59.32)	101 (57.06)	99 (56.25)	113 (63.48)	
Vascular risk factors, (%)					
Current Drinking Alcohol	46 (25.99)	42 (23.73)	53 (30.11)	43 (24.16)	0.512
Current Smoking	53 (29.94)	54 (30.51)	53 (30.11)	52 (29.21)	0.996
History of diabetes mellitus	30 (16.95)	29 (16.38)	55 (31.25)	81 (45.51)	<0.001
History of hypertension	120 (67.80)	123 (69.49)	138 (78.41)	139 (78.09)	0.037
History of atrial fibrillation	48 (27.12)	40 (22.60)	35 (19.89)	27 (15.17)	0.045
History of coronary heart disease	26 (14.69)	29 (16.38)	24 (13.64)	25 (14.04)	0.898
Clinical assessment, (%)					
PAO	56 (31.64)	62 (35.03)	63 (35.80)	69 (38.76)	0.574
NIHSS group					0.135
Mild (0–4)	85 (48.02)	70 (39.55)	73 (41.48)	65 (36.52)	
Moderate (5–10)	45 (25.42)	53 (29.94)	51 (28.98)	44 (24.72)	
Moderately Severe (11–20)	40 (22.60)	48 (27.12)	38 (21.59)	57 (32.02)	
Severe (≥21)	7 (3.95)	6 (3.39)	14 (7.95)	12 (6.74)	
Stroke subtype (TOAST)					<0.001
LAA	56 (31.64)	74 (41.81)	76 (43.18)	97 (54.49)	
CE	74 (41.81)	57 (32.20)	43 (24.43)	37 (20.79)	
SAO	41 (23.16)	39 (22.03)	41 (23.30)	39 (21.91)	
Other/Unknown	6 (3.39)	7 (3.95)	16 (9.09)	5 (2.81)	
OTT					0.380
<3 h	123 (69.49)	133 (75.14)	119 (67.61)	121 (67.98)	
3–4.5 h	54 (30.51)	44 (24.86)	57 (32.39)	57 (32.02)	
Medication use, (%)					
Antiplatelet therapy					0.543
Never use	23 (12.99)	17 (9.60)	14 (7.95)	20 (11.24)	
New Initiation Post-Stroke	121 (68.36)	130 (73.45)	138 (78.41)	129 (72.47)	
Use prior to stroke	33 (18.64)	30 (16.95)	24 (13.64)	29 (16.29)	
Anticoagulation therapy					0.261
Never use	119 (67.23)	122 (68.93)	124 (70.45)	134 (75.28)	
New initiation post-stroke	46 (25.99)	51 (28.81)	45 (25.57)	38 (21.35)	
Use prior to stroke	12 (6.78)	4 (2.26)	7 (3.98)	6 (3.37)	
Functional outcome, (%)					<0.001
Favorable	134 (75.71)	133 (75.14)	112 (63.64)	98 (55.06)	
Unfavorable	43 (24.29)	44 (24.86)	64 (36.36)	80 (44.94)	

High-sensitivity CTI, high-sensitivity C-reactive protein-triglyceride-glucose index; NIHSS, National Institutes of Health Stroke Scale; OTT, onset to treatment time; PAO, proximal arterial occlusion; TOAST, Trial of Org 10,172 in Acute Stroke Treatment; LAA, large-artery atherosclerosis; CE, cardioembolism; SAO, small-artery occlusion.

Comparisons across high-sensitivity CTI quartiles were performed using ANOVA, Kruskal-Wallis test, χ^2^ test, or Fisher’s exact test as appropriate.

### Association between high-sensitivity CTI and outcome

Figure 1 illustrated the violin plots of high-sensitivity CTI levels between the favorable functional outcome group and the unfavorable functional outcome group. An unadjusted ordinal logistic regression was performed was performed to test the trend across high-sensitivity CTI quartiles for the full distribution of mRS scores. A significant shift toward poorer functional outcomes was observed with rising CTI quartiles among elderly AIS patients receiving intravenous thrombolysis (*P* for trend < 0.001; Figure 2).

**Figure 1:**
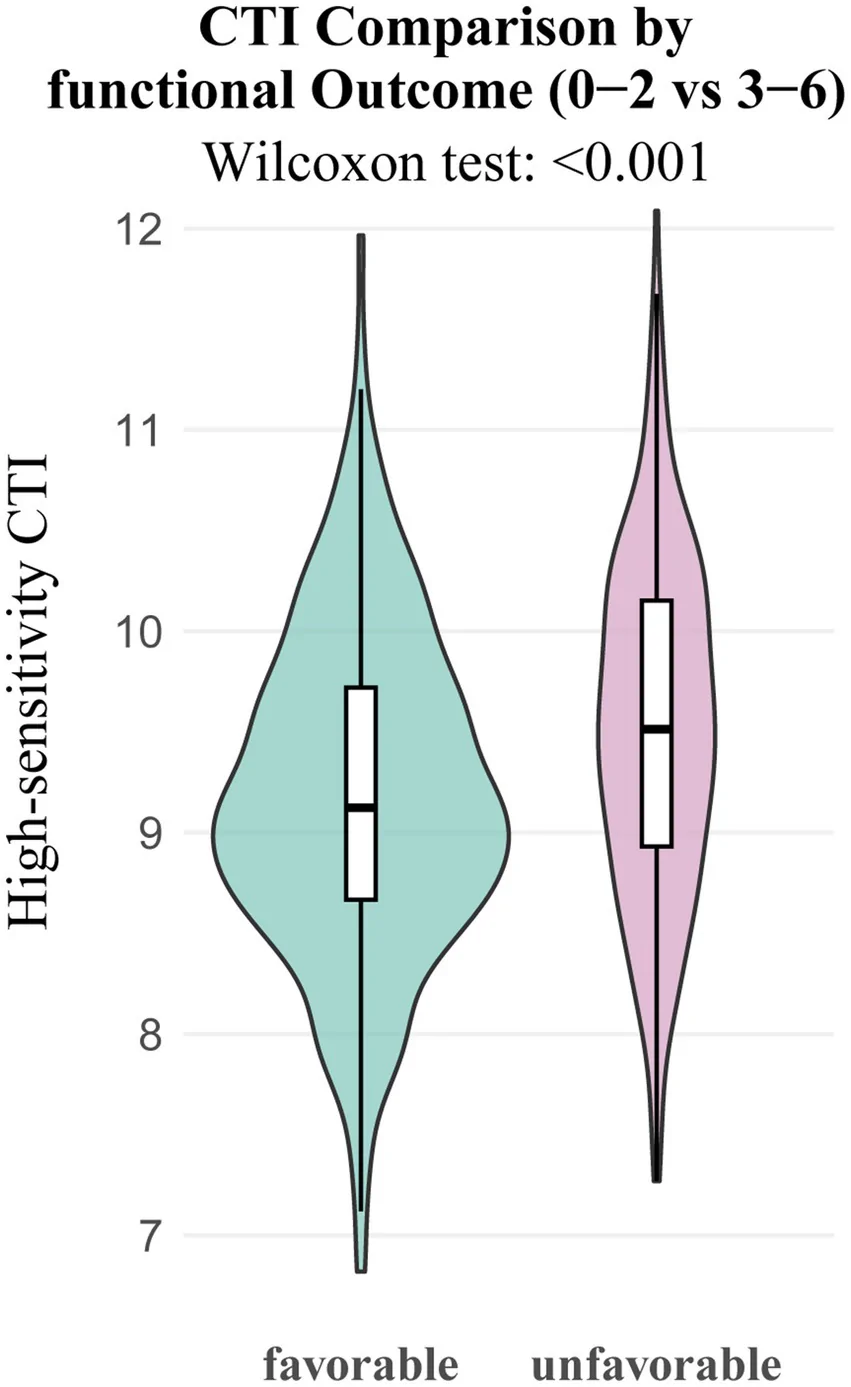
The violin plot in distribution of high-sensitivity CTI between the favorable functional outcome group and the unfavorable functional outcome group.

**Figure 2:**
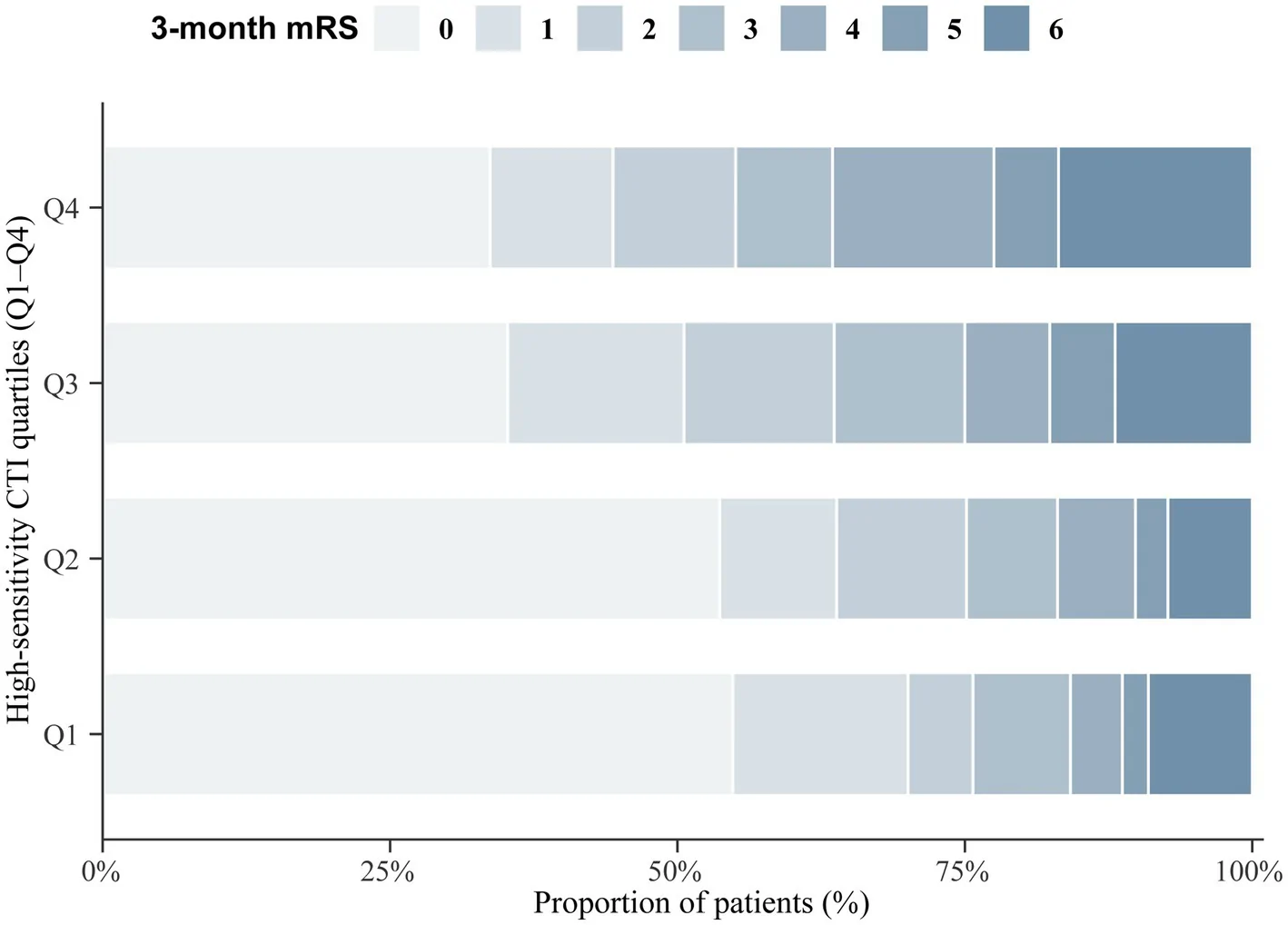
Distribution of 3-month modified Rankin Scale score according to high-sensitivity CTI quartiles among elderly acute ischemic stroke patients undergoing IVT by unadjusted ordinal logistic regression.

Figure 3 showed the results of logistic regression. In the model 1, the levels of high-sensitivity CTI were associated with unfavorable functional outcome at 3 months in elderly AIS patients undergoing IVT (odds ratio [OR], 1.61; 95% confidence interval [CI], 1.32–1.96, *p* < 0.001), when we serve the levels of high-sensitivity CTI as continuous variables. What is more, the third quartile (OR, 1.78; 95% CI, 1.12–2.82, *p* = 0.014) and fourth quartile (OR, 2.54; 95% CI, 1.62–4.00, *p* < 0.001) of high-sensitivity CTI (first quartile used as the reference value) were also linked to unfavorable functional outcome. Additionally, similar results were found in model 2 and model 3. In model 2, the levels of high-sensitivity CTI, as continuous variables, were related to unfavorable functional outcome at 3 months (OR, 1.69; 95% CI, 1.38–2.07, *p* < 0.001). The third quartile (OR, 1.82; 95% CI, 1.13–2.91, *p*  =0.013) and fourth quartile (OR, 2.85; 95% CI, 1.79–4.55, *p* < 0.001) of high-sensitivity CTI (first quartile used as the reference value) were linked to unfavorable functional outcome. In Model 3, the levels of high-sensitivity CTI, as continuous variables, were related to unfavorable functional outcome at 3 months (OR, 1.64; 95% CI, 1.28–2.11, *p* < 0.001). The third quartile (OR, 1.93; 95% CI, 1.10–3.40, *p* = 0.023) and fourth quartile (OR, 2.62; 95% CI, 1.48–4.64, *p* < 0.001) of high-sensitivity CTI (first quartile used as the reference value) were linked to unfavorable functional outcome.

**Figure 3:**
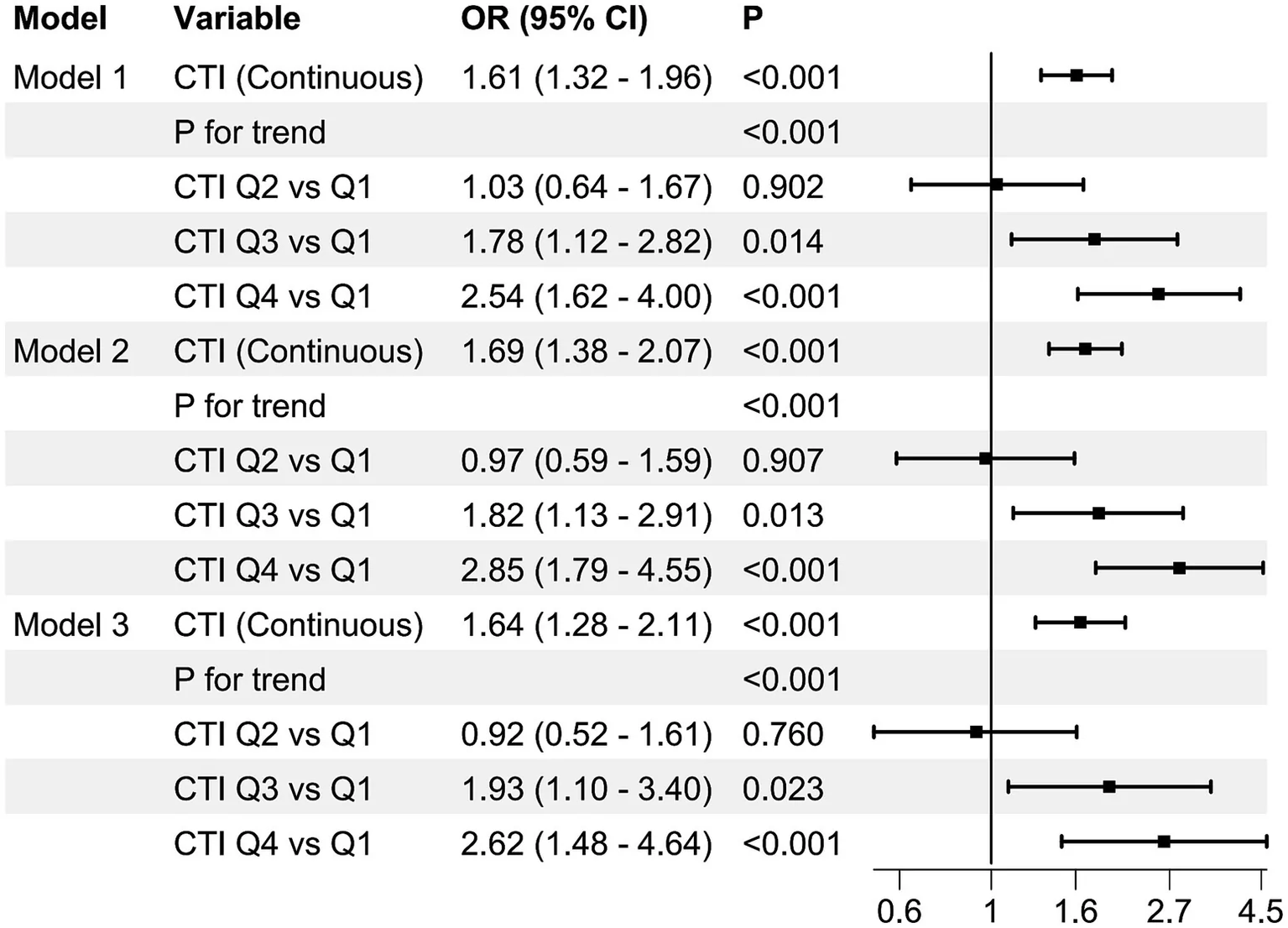
OR and 95% CI of unfavorable functional outcome according to high-sensitivity CTI among elderly acute ischemic stroke patients undergoing IVT. Model 1: Crude model; Model 2: Adjusted for age and gender, current smoking and current drinking alcohol; Model 3: Adjusted for age and gender, current smoking, current drinking alcohol, history of hypertension, history of diabetes mellitus, history of atrial fibrillation, history of coronary artery disease, antiplatelet, anticoagulation, NIHSS group, OTT, PAO and stroke subtype.

### RCS analysis

According to the results of RCS analysis (Figure 4A), high-sensitivity CTI was associated with unfavorable 3-month functional outcome after IVT (*P* for overall < 0.001), and no statistically significant nonlinear association was detected (*P* for nonlinearity = 0.743).

**Figure 4:**
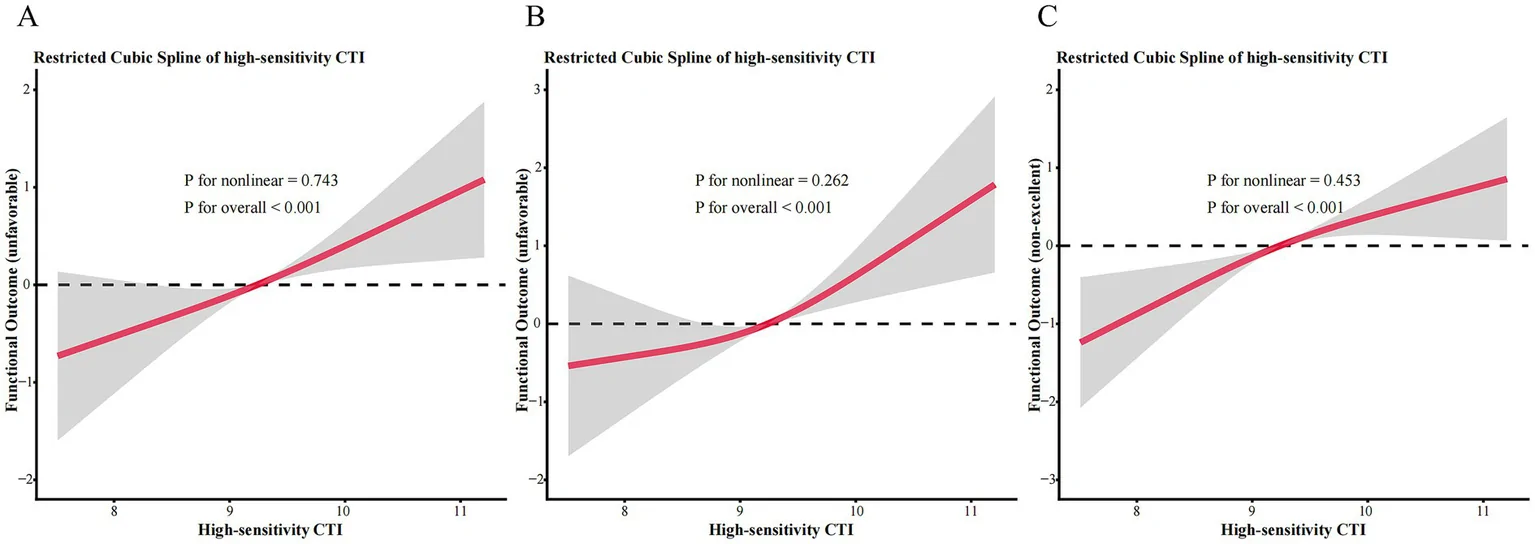
Relationship of high-sensitivity CTI with functional outcome in elderly acute ischemic stroke patients undergoing IVT. **(A)** Relationship of high-sensitivity CTI with unfavorable functional outcome (mRS 3–6). **(B)** Relationship of high-sensitivity CTI with unfavorable functional outcome (mRS 3–6) excluding PAO patients. **(C)** Relationship of high-sensitivity CTI with non-excellent functional outcome (mRS 2–6).

### Subgroup analyses

Subgroup analyses demonstrated that higher high-sensitivity CTI was consistently associated with elevated risk of unfavorable functional outcome across all examined strata (Figure 5). No significant interactions were found between high-sensitivity CTI and these subgroup factors (all *P* for interaction > 0.05).

**Figure 5:**
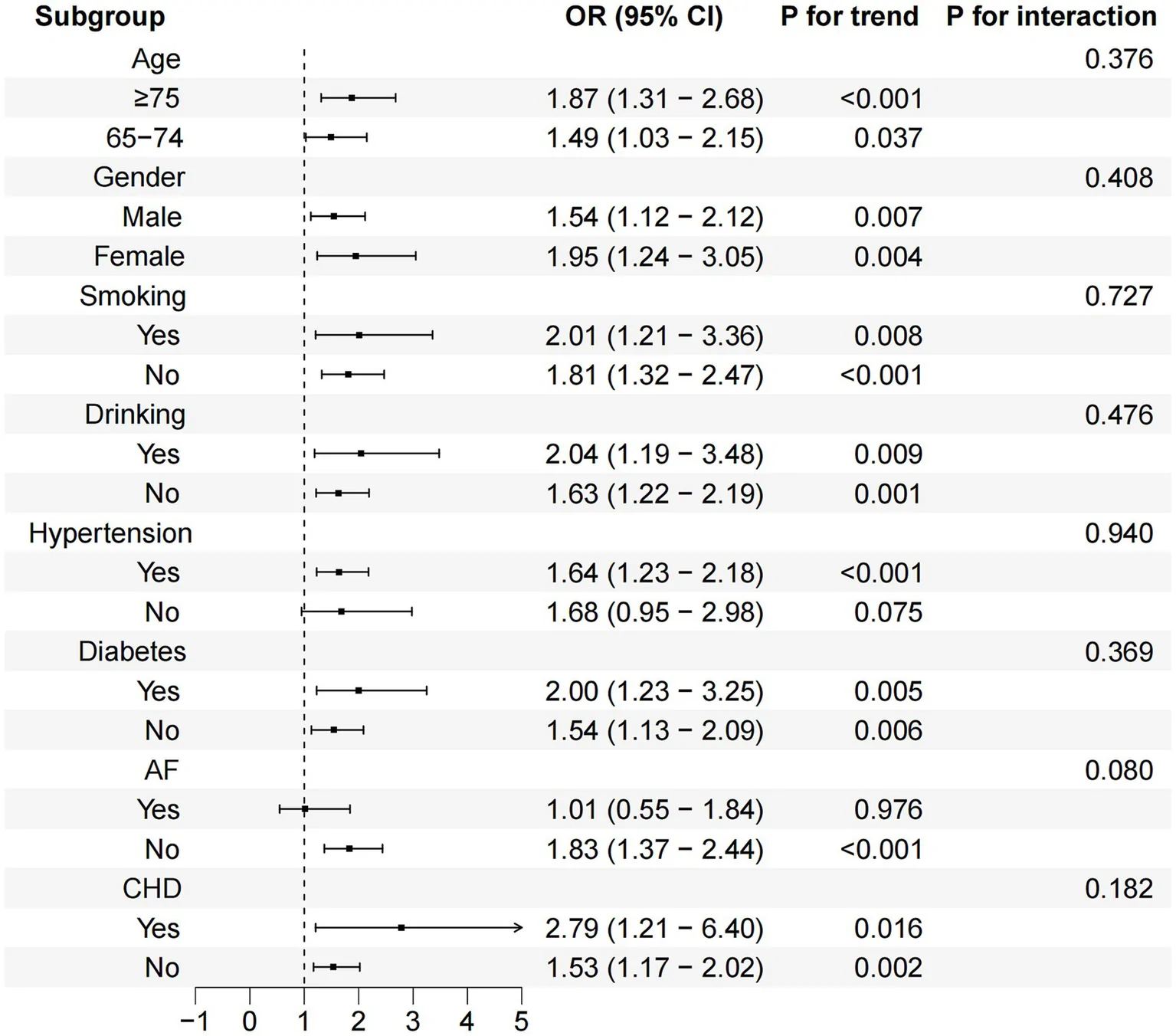
Subgroup analysis of the association between high-sensitivity CTI and unfavorable functional outcome.

### ROC curve analysis

To further evaluate the discriminatory capacity of high-sensitivity CTI for unfavorable 3-month functional outcomes after IVT, we performed ROC curve analysis (Figure 6A). The AUC of high-sensitivity CTI was 0.613 (95% CI 0.568–0.657), indicating modest discriminatory capacity for post-thrombolysis unfavorable functional outcome. The optimal cutoff value of high-sensitivity CTI derived from the maximum Youden’s index was 9.299, yielding a sensitivity of 0.610 and specificity of 0.591 at this threshold.

**Figure 6:**
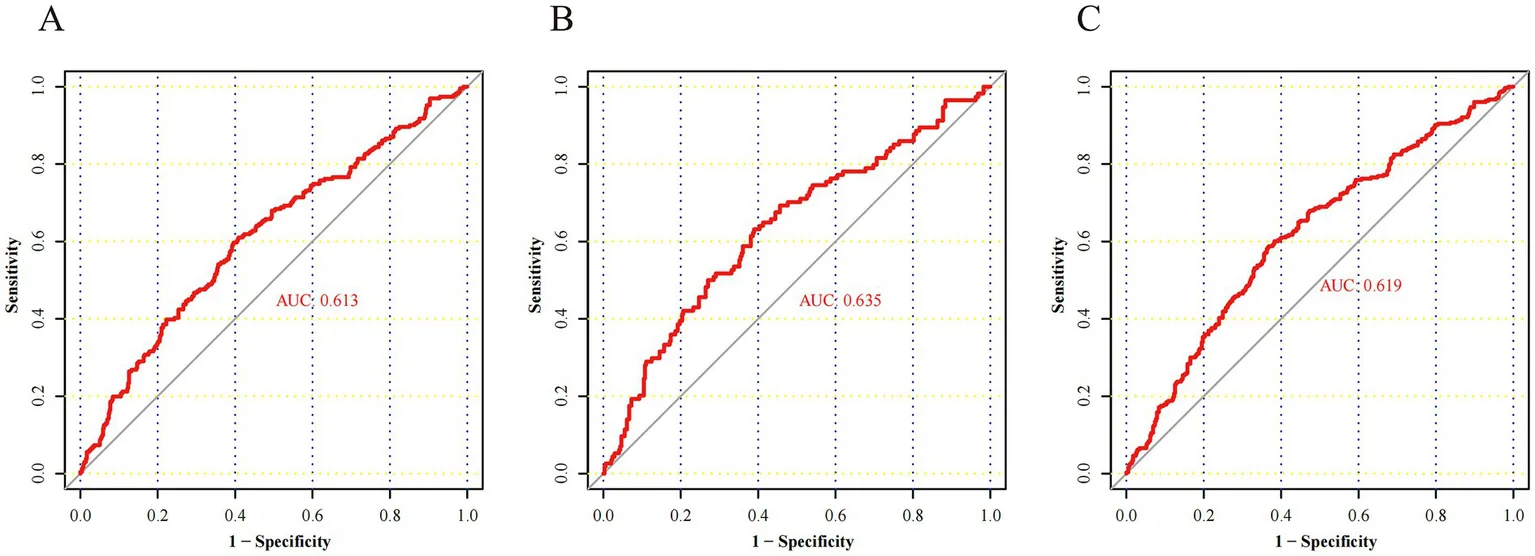
ROC for high-sensitivity CTI for discrimination of functional outcome. **(A)** ROC for high-sensitivity CTI for discrimination of unfavorable functional outcome (mRS 3–6). **(B)** ROC for high-sensitivity CTI for discrimination of unfavorable functional outcome (mRS 3–6) excluding PAO patients. **(C)** ROC for high-sensitivity CTI for discrimination of non-excellent functional outcome (mRS 2–6).

### Sensitivity analyses

We excluded the patients with PAO and performed the sensitivity analyses. As shown in Figure 7, after the adjustment for model 3, the fourth quartile (OR, 3.15; 95% CI, 1.47–6.75, *p*  =  0.003) of high-sensitivity CTI (first quartile used as the reference value) were also related with unfavorable functional outcome in elderly AIS patients undergoing IVT. In addition, levels of high-sensitivity CTI, as continuous variables, were also linked to the unfavorable functional outcome in this model (OR, 1.92; 95% CI, 1.36–2.71, *p* < 0.001). Moreover, we observed an overall association between high-sensitivity CTI and unfavorable functional outcome (*P* for overall < 0.001), while no statistically significant nonlinear association was detected (*P* for nonlinearity = 0.262; Figure 4B). AUC of high-sensitivity CTI to discriminate post-thrombolysis unfavorable functional outcome was 0.635 (a sensitivity of 0.632 and a specificity of 0.610; Figure 6B). We also served non-excellent functional outcome (mRS 2–6) as the study outcome. In the Model 3, the third quartile (OR, 3.04; 95% CI, 1.76–5.26, *p* < 0.001) and fourth quartile (OR, 3.40; 95% CI, 1.94–5.96, *p* < 0.001) of high-sensitivity CTI (first quartile used as the reference value) were associated with non-excellent functional outcome (Figure 8). Levels of high-sensitivity CTI, as continuous variables, were also related with the non-excellent functional outcome in this model (OR, 1.75; 95% CI, 1.37–2.23, *p* < 0.001). What is more, we observed an overall association between high-sensitivity CTI and the non-excellent functional outcome (*P* for overall < 0.001), while no statistically significant nonlinear association was detected (*P* for nonlinearity = 0.453; Figure 4C). AUC of high-sensitivity CTI to discriminate post-thrombolysis non-excellent functional outcome was 0.619 (a sensitivity of 0.587 and a specificity of 0.632; Figure 6C).

**Figure 7:**
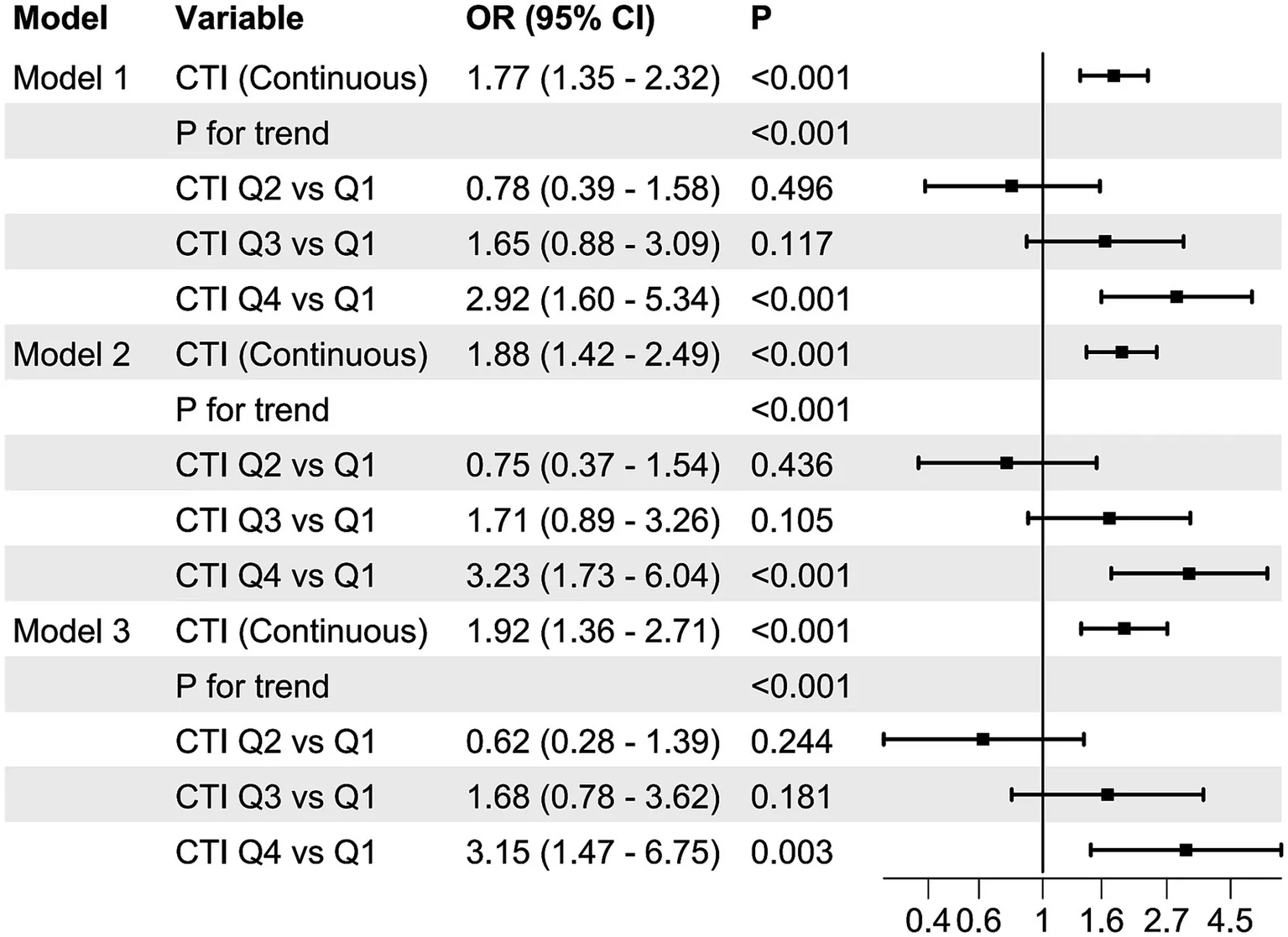
OR and 95% CI of unfavorable functional outcome according to high-sensitivity CTI among elderly acute ischemic stroke patients undergoing IVT, excluding the patients with PAO. Model 1: Crude model; Model 2: Adjusted for age and gender, current smoking and current drinking alcohol; Model 3: Adjusted for age and gender, current smoking, current drinking alcohol, history of hypertension, history of diabetes mellitus, history of atrial fibrillation, history of coronary artery disease, antiplatelet, anticoagulation, NIHSS group, OTT and stroke subtype.

**Figure 8:**
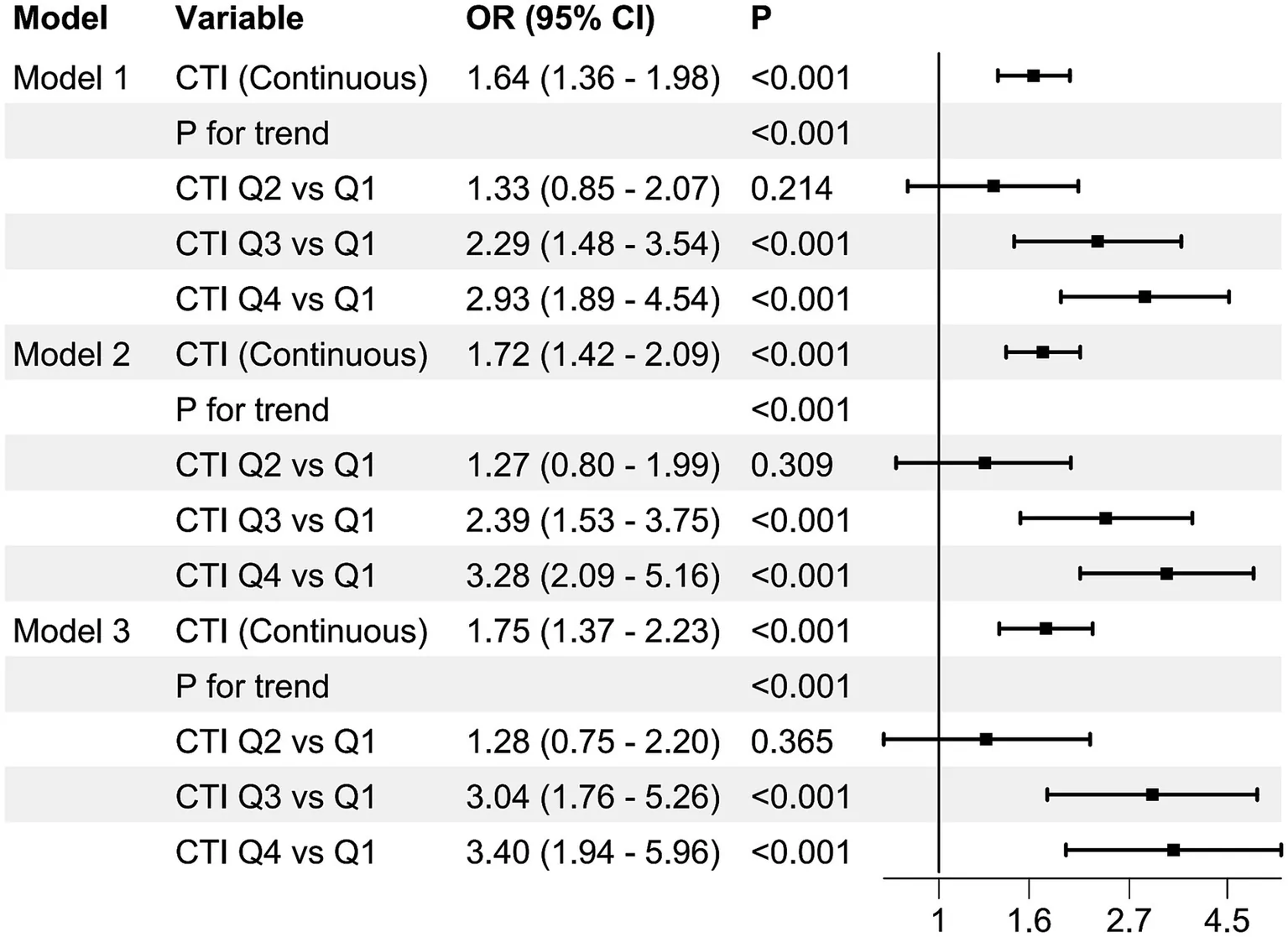
OR and 95% CI of non-excellent functional outcome (mRS 2–6) according to high-sensitivity CTI among elderly acute ischemic stroke patients undergoing IVT. Model 1: Crude model; Model 2: Adjusted for age and gender, current smoking and current drinking alcohol; Model 3: Adjusted for age and gender, current smoking, current drinking alcohol, history of hypertension, history of diabetes mellitus, history of atrial fibrillation, history of coronary artery disease, antiplatelet, anticoagulation, NIHSS group, OTT, PAO and stroke subtype.

## Discussion

Our study focused on the link between high-sensitivity CTI levels and the functional outcomes in elderly AIS patients who received IVT. The results of our study showed that the elevated levels of high-sensitivity CTI might be related with the increased probability of the unfavorable functional outcomes in elderly AIS patients treated with IVT in diverse regression models. Thus, these findings imply that the levels of high-sensitivity CTI may have a particular association with the post-thrombolysis prognosis of elderly AIS patients.

It is reported that TyG, calculated from triglycerides and blood glucose, is one of reliable biomarkers of IR (37, 38). IR is associated with metabolic diseases, for instance, hyperglycemia and dyslipidemia. These diseases mentioned above are proved to be the risk factors of ischemic stroke, the frequent illness in the elderly. The results of an 11-year follow-up study implied that high levels of both baseline and long-term updated cumulative average TyG index might be able to predict ischemic stroke, independently (39). The research utilizing data from the China National Stroke Registry II, indicated that TyG could be related to the recurrence of ischemic stroke and all-cause death in AIS patients with type-2 diabetes mellitus (40). Moreover, TyG was identified as an independent risk factor in critically ill patients with first-ever stroke (18). In addition, many studies have confirmed the role of inflammatory response in the pathophysiological mechanism of stroke (19–21, 41, 42). High-sensitivity CRP is considered as one of convenient and simple inflammatory biomarkers. One previous study showed that high-sensitivity CRP might be a risk factor for ischemic stroke and retinal artery occlusion (43). The analysis of UK Biobank data revealed that elevated and remaining high levels of CRP were related to both any stroke as well as ischemic stroke (44). Wang et al. found that high-sensitivity CRP could serve as the biomarker of stroke recurrence (45). CRP also is report to be linked to carotid restenosis in elderly AIS patients undergoing interventional surgery (46). Hence, CTI, which integrates the advantages of CRP and TyG, may be able to play a critical role in the mechanism of ischemic stroke. One recent large-scale study, analyzing the data from China Health and Retirement Longitudinal Study (CHARLS), has identified that elevated levels of CTI may be linked to an increased risk of stroke in people with normal glucose regulation or prediabetes mellitus (24), which perhaps highlights the potential role of CTI in stroke. Another study based on CHARLS showed the relationship between CTI levels and the risk of stroke in the patients with hypertension (47).

The violin plot suggested that there was a significant difference in the levels of high-sensitivity CTI between the favorable functional outcome group and the unfavorable functional outcome group. Owing to the intricateness of the prognosis of elderly AIS patients, we developed several regression models and included high-sensitivity CTI as either a categorical variable or a continuous variable into these regression models, all of which depicted that the increased levels of high-sensitivity CTI might be linked to the worse prognosis of elderly AIS patients who received IVT. Subgroup analyses showed that the association of high-sensitivity CTI with post-thrombolysis functional outcome was qualitatively consistent across different clinical strata, with no significant subgroup interactions detected. Furthermore, we preformed the following sensitivity analyses: excluding the elderly AIS patients with PAO, or regarding mRS score > 1 as the outcome. After these sensitivity analyses, we observed analogous results, which denoted that further research is needed in the future to explore the role of high-sensitivity CTI in the prognosis of elderly AIS patients undergoing IVT.

There are several potential limitations in this study. First, given that all participants in our study were Chinese elderly patients undergoing IVT, the relationship between the levels of high-sensitivity CTI and functional outcome in elderly AIS patients treated with IVT may not be able to be generalized to non-Chinese populations. Second, some risk factors that may be linked to post-thrombolysis functional outcome, such as stromal cell-derived factor (SDF)-1 (48) and brain-derived tau (49), were unavailable in this study. These biomarkers might serve as potential independent predictors for functional outcome in elderly AIS patients treated with IVT. Third, the levels of high-sensitivity CTI might be able to vary during hospitalization. Our future research needs dynamic examination of high-sensitivity CTI. Fourth, considerable patient exclusions may introduce potential selection bias, and categorization of NIHSS and OTT could further cause information loss and residual confounding, as the adjustment for post-baseline in-hospital antiplatelet and anticoagulant therapies which may bring over-adjustment bias. Fifth, high-sensitivity CTI only showed modest discriminatory performance for functional outcome. In addition, although all centers adhered to standardized routine clinical laboratory quality-control procedures for biomarker testing, measurements were undertaken on distinct platforms with different reagents, which may introduce inter-center measurement heterogeneity. Despite these limitations mentioned above, the present analysis adds evidence regarding the association between high-sensitivity CTI and 3-month functional outcome specifically among elderly AIS patients treated with IVT, a high-risk subgroup that has received limited dedicated investigation (50).

To sum up, our research indicated that high-sensitivity CTI, a readily accessible biomarker, was associated with functional outcomes in older AIS patients receiving IVT. To confirm these results about this conclusion, further research will be needed.

## Data Availability

The raw data supporting the conclusions of this article will be made available by the authors, without undue reservation.

## Footnotes

1
http://www.R-project.org/
